# Aripiprazole Augmentation in the Treatment of Military-Related PTSD with Major Depression: a retrospective chart review

**DOI:** 10.1186/1471-244X-11-86

**Published:** 2011-05-17

**Authors:** J Don Richardson, Deniz Fikretoglu, Aihua Liu, Diane McIntosh

**Affiliations:** 1 Operational Stress Injury Clinic, St. Joseph's Health Care London - Parkwood Hospital, London, Ontario, Canada; 2 National Centre for Operational Stress Injuries, Veterans Affairs Canada, Montreal, Quebec, Canada; 3 Department of Psychiatry, Schulich School of Medicine and Dentistry, University of Western Ontario, Canada; 4 Defense Research and Development Canada, Toronto, Canada; 5 Douglas Mental Health University Institute, McGill University, Montreal, Quebec, Canada; 6 Department of Epidemiology, Biostatistics and Occupational Health, McGill University, Montreal, Quebec, Canada; 7 Department of Psychiatry, University of British Columbia, Vancouver, BC, Canada

## Abstract

**Background:**

In this chart review, we attempted to evaluate the benefits of adding aripiprazole in veterans with military-related PTSD and comorbid depression, who had been minimally or partially responsive to their existing medications.

**Methods:**

A retrospective chart review of patients who received an open-label, flexible-dose, 12- week course of adjunctive aripiprazole was conducted in 27 military veterans meeting DSM-IV criteria for PTSD and comorbid major depression. Concomitant psychiatric medications continued unchanged, except for other antipsychotics which were discontinued prior to initiating aripiprazole. The primary outcome variable was a change from baseline in the PTSD checklist-military version (PCL-M) and the Beck Depression Inventory (BDI-II).

**Results:**

PTSD severity (Total PCL scores) decreased from 56.11 at baseline to 46.85 at 12-weeks (p < 0.0001 from Wilcoxon signed rank test) and the depression severity decreased from 30.44 at baseline to 20.67 at 12-weeks (p < 0.0001 from Wilcoxon signed rank test). Thirty seven percent (10/27) were considered responders, as defined by a decrease in total PCL scores of at least 20 percent and 19% (5/27) were considered as responders as defined by a decrease in total BDI score of at least 50%.

**Conclusions:**

The addition of aripiprazole contributed to a reduction in both PTSD and depression symptomatology in a population that has traditionally demonstrated poor pharmacological response. Further investigations, including double-blind, placebo-controlled studies, are essential to confirm and further demonstrate the benefit of aripiprazole augmentation in the treatment of military related PTSD.

## Background

Military-related posttraumatic stress disorder (PTSD) is a serious psychiatric condition often resulting from combat duty in the current wars in Afghanistan and Iraq [1] and past peacekeeping and humanitarian missions [2-4]. Patients with PTSD present with four symptom clusters: reexperiencing of the traumatic event(s), avoidance of reminders and emotional numbing (which are grouped together as one symptoms cluster in DSM-IV, but are seen as distinct and will likely be denoted as such in DSM-V), and hyperarousal symptoms [5,6]. Recent estimates of the prevalence of PTSD in various military and veteran populations have varied from a low of 4.8% in UK military members [7], to 10.3% in Canadian peacekeeping veterans, [8] and 11.2 - 17.1% in U.S. military members returning from the deployments to Iraq and Afghanistan [9]. Military-related PTSD is associated with severe psychosocial dysfunction [10,11].

The therapeutic response to pharmacological interventions for military-related PTSD is often disappointing [10,12-16]. PTSD often presents with co-morbidities such as depression and substance abuse or dependence [17,18]. Amongst veterans, the comorbidity rates may be much higher than in other populations [19,20]. Studies have also demonstrated that veterans with chronic, military-related PTSD often present with significant comorbid psychotic features [21,22] which may contribute to the severe psychosocial dysfunction in this population [10,11].

Selective Serotonin Reuptake Inhibitors (SSRIs) and Selective Serotonin/Norepinephrine Reuptake Inhibitors (SNRIs) have the most empirical evidence for efficacy in the treatment of PTSD and are usually considered first-line treatment [5,16,23]. SSRIs and SNRIs are also effective agents for the treatment of co-morbid mood and anxiety disorders commonly associated with PTSD. However, the lack of efficacy of antidepressant monotherapy, especially in male combat veterans, [24,25] has led to the frequent use of combination strategies, especially the addition of antipsychotics, in many treatment guidelines for treatment-resistant PTSD [5,26].

The benefit of adding a second-generation antipsychotic, such as risperidone, quetiapine, or olanzapine for the treatment of PTSD in combination with a primary antidepressant has been suggested in numerous small studies, including a few randomized controlled trials [27-30]. These agents appear beneficial in managing hyperarousal symptoms such as hypervigilance and irritability, as well as severe dissociative symptoms [16]. There is also evidence for the addition of aripiprazole, quetiapine, risperidone, and olanzapine for treatment-resistant depression, [31] which often presents as a complicating factor in military-related, chronic PTSD. More recently, the efficacy of aripiprazole in the treatment of PTSD has been demonstrated in three preliminary open-label studies in veteran populations, both as a monotherapy and as an adjunctive treatment [32-34].

Aripiprazole is a novel antipsychotic with partial agonist activity at D2 receptors and 5HT1A receptors, and antagonist activity at 5-HT2A receptors [35]. Aripiprazole is reported to have less risk for extrapyramidal side effects than traditional antipsychotics [36,37] and has been demonstrated to be effective in treatment-resistant depression [38,39].

Based on the positive results of aripiprazole for both treatment-resistant depression and treatment-resistant PTSD, and on its unique pharmacology, we hypothesized that aripiprazole would be efficacious for treatment-resistant military-related PTSD with comorbid major depression. To reflect general clinical practice, we conducted a retrospective file review to examine the benefits of adding aripiprazole in veterans with military-related PTSD and comorbid depression, who had been minimally or partially responsive to their existing medications. To our knowledge, this is the first attempt to examine the efficacy of aripiprazole to treat both chronic PTSD and comorbid depression in a sample of veterans with chronic military-related PTSD where all of the participants had comorbid major depression.

## Methods

### Participants and Procedure

Participants consisted of a sample of 27 out of 123 consecutive veterans who consented to try aripiprazole augmentation between November 2009 and August 2010. All patients were receiving outpatient psychiatric care at a clinic specializing in the assessment and treatment of military-related psychiatric conditions. The clinic follows a standardized assessment and treatment protocol; the standardized assessments included the PTSD Checklist-Military Version (PCL-M), [40] Beck Depression Inventory (BDI), [41] and Medical Outcomes Study (MOS) 36-item Short-Form Health Survey (SF-36), [42] administered at intake and at each follow-up appointment over the course of treatment. In addition to providing psychoeducation, the standard psychiatric treatment at the clinic includes symptom management, treatment of comorbid disorders, and management of functional impairment [5]. Participants were prescribed aripiprazole after demonstrating minimal or partial response to their existing antidepressant and/or minimal or partial response to other antipsychotic augmentation strategies. All subjects received a comprehensive psychiatric evaluation and laboratory tests (complete blood count with white count differential, serum electrolytes, glucose, creatinine, blood urea nitrogen, liver function tests, and lipid profile). The initial dose of aripiprazole was 2 to 5 mg daily, with further dose titrations based on tolerability and clinical response, up to a maximum of 30 mg daily. Efficacy and adverse effects were assessed and recorded at each follow-up appointment (bi-weekly for the first month and then monthly). Antidepressants, anxiolytics, and mood stabilizers were allowed but had to be kept at a constant dose during the 12- weeks treatment phase. As we were specifically interested in examining the benefits of aripiprazole augmentation in veterans with military-related PTSD, patients not prescribed aripiprazole augmentation were excluded from this chart review. For patients who were already prescribed an antipsychotic, the antipsychotic was discontinued prior to the initiation of aripiprazole.

The sample was derived from a retrospective chart review with approval from the Office of Research Ethics of the University of Western Ontario. Consent from each patient was not obtained specifically for this chart review. However, as part of the initial orientation to the clinic, all patients are asked to provide written consent to participate in research. All patients met the DSM-IV criteria for PTSD and comorbid major depressive disorder. To maximize the generalizability of this evaluation to usual "clinical practice," all comorbid physical and psychiatric conditions were included in the chart review.

### Instruments

To diagnose PTSD, the Clinician-Administered PTSD Scale (CAPS) [43] was administered by a trained clinician and the diagnosis of major depressive disorder was determined using the Patient Health Questionnaire (PHQ-9)[44] and the psychiatric interview according to the *Diagnostic and Statistical Manual of Mental Disorders, Fourth Edition *(DSM-IV) criteria [6]. To assess change in PTSD symptoms with treatment, the PCL-M [40] was used. Similarly, the BDI [45,46] was used to assess change in depressive symptoms as a result of treatment. The SF-36 [47] was used to assess health related quality of life (HRQol). The SF-36 measures functional impairment in eight domains or subscales; the mental health sub scales can be collapsed into the Mental Component Summary (MCS) Score reflecting overall mental health [48]. The PCL-M, the BDI, and the SF-36 were all administered at each follow-up appointment including at intake (t1) and at 1-, 2-, and 3-month follow-ups (t2, t3, and t4) over the course of treatment. The total PCL-M and the total BDI were the primary efficacy variables; the total PCL-M re-experiencing, avoidance/numbing, and hyperarousal subscale scores served as secondary efficacy variables.

### Analysis

The LOCF data for each visit included the data recorded at that visit or carried forward from the last visit. For the primary outcome, the Wilcoxon signed rank test and effect sizes (Cohen's d) was used to determine the statistical significance of the change from baseline total PCL-M and Total BDI score at each follow-up time point (t2, t3, t4). For the three secondary outcome measures (the PCL-M reexperiencing, avoidance/numbing, and hyperarousal subscale scores), only the effects at t4 were examined. To correct for multiple comparisons, we used Hochberg step-up multiple comparisons procedure [49].

For each of the two main outcomes, we identified responders on our two outcome measures using criteria established in existing literature. More specifically, on the PCL, we identified those with at least a 20% reduction in their total PCL scores as responders; [33] this is in keeping with prior treatment efficacy and more specifically, prior Aripiprazole treatment efficacy studies for PTSD [33]. On the BDI, we identified those with at least a 50% reduction in their symptom scores as responders [50].

## Results

### Demographics and Clinical Characteristics

Demographic and clinical characteristics of the sample are presented in Table 1. Of the 27 participants, almost all were men (*n *= 26, 96.3%), with an average age of 39.36 years (*SD *= 5.98) and an average of 10.26 years (*SD *= 7.38) with PTSD symptoms. At intake, the majority (*n = 20*, 74.1%) of the sample had been released from the military. Years of military service averaged 15.04 years (*SD *= 7.41). Almost all (*n *= 26, 96.3%) had exposure to combat or to a war zone during their deployment and the most common deployments reported were Afghanistan (*n *= 9, 33.3%), the former Yugoslavia (*n *= 8, 29.6%), and Africa (Somalia, Rwanda, Eatrea, and Sierra Leone) (*n *= 5, 18.5%). All patients were taking an antidepressant prior to initiating aripiprazole, most commonly a norepinephrine dopamine reuptake inhibitor (NDRI) (*n *= 16, 59.3%), followed by noradrenergic specific serotonergic antidepressants (NaSSA) (*n *= 12, 44.4%); serotonin norepinephrine reuptake inhibitor (SNRI) (*n *= 11, 41.7%), and selective serotonin reuptake inhibitor (SSRI) (*n *= 10, 37%). Most patients (*n *= 14, 51.9%) were taking two antidepressant and an additional 14.8% (*n *= 4) were taking three antidepressant. In addition to an antidepressant, 8 patients (30.77%) were prescribed an anticonvulsant, 5 patients (19.23%) were prescribed a stimulant, and 4 patients (15.38%) were prescribed a hypnotic.

**Table 1:** Baseline characteristics of the sample

Demographic and clinical variables	n (%) or mean (sd)
Age	39.36 (6.09)


Sex	
Male	26 (96.30%)
Female	1 (3.70%)


Canadian Forces (CF) status	
Released	20 (74.07%)
Still serving	7 (25.93%)


Current Work Status	
Unemployed	17 (62.96%)
Working for Pay	6 (22.22%)
On Sick Leave from Work	4 (14.81%)


Duration of illness	10.26 (7.38)


SF-36 Mental component score	20.22 (10.11)
SF-36 Physical component score	32.48 (15.06)

Twenty-seven patients had at least one post-baseline efficacy evaluation thus were included in the efficacy analysis. At intake, all participants met full criteria for PTSD based on the CAPS interview. The average duration of PTSD symptoms was 10.26 years (*SD *= 7.38), suggesting a chronic course for this sample. Intake scores on the PCL-M and the BDI averaged 56.11(*SD *= 12.66) and 30.44 years (*SD *= 7.86), respectively. On the PCL-M, a score of 50 is the conventional cut-off score for a positive screen for PTSD in veteran populations; [51] on the BDI, scores above 29 are considered indicative of severe depression [52]. SF-36 MCS and PCS were 20.22 (SD = 10.11) and 32.48 (SD = 15.06), respectively, indicating significant impairment in scales measuring both mental and physical functioning.

### Dose and Tolerability

The final average dose of aripiprazole was 12.40 (SD = 4.35) mg daily. The mean value of weight decrease from baseline (mean = 99.25 kg, SD = 13.35) to the final visit is 1.05 kg (SD = 4.95). Only two patients discontinued the aripiprazole; one patient due to non-response and one due to intolerable restlessness. The remaining 25 patients tolerated the aripiprazole. The most common side effects reported were insomnia (N = 5/27, 18.5%); agitation/irritability (N = 4/27, 14.8%); restlessness (N = 3/27, 11.1%); and fatigue (N = 2/27, 7.4%). The good tolerability was likely related to lower starting doses (2 mg daily) and slow titration (increasing the dose by 2-5 mg every two weeks).

### Treatment Efficacy

**Figure 1:**
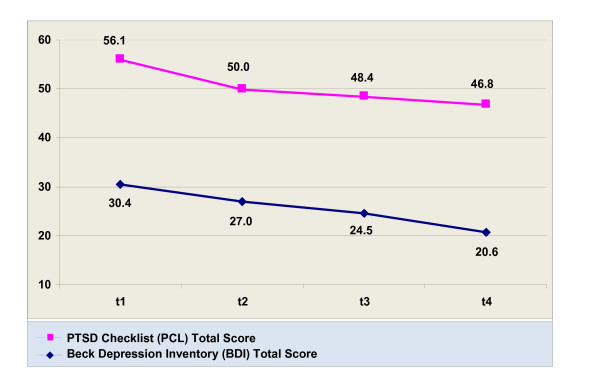
**Change in PCL and BDI total score from baseline (t1) to 3 months (t4) for patients receiving aripiprazole**.

Results from Wilcoxon signed rank tests showed significant decreases between baseline and each of the three follow-ups for the BDI and the PCL, as well as the last visit for the PCL Reexperiencing, Avoidance, and Hyperarousal subscales (Table 2), even after multiple testing correction. The total PCL score decreased from 56.11 (*SD *= 12.66) at baseline to 46.85 (*SD *= 13.53) at three months and the total BDI score 30.44 (*SD = *7.85) at baseline to 20.67 (*SD *= 10.05) at three months, figure 1. Effect sizes and changes in the clinical outcome variables from intake to the final visit are reported in Table 3.

**Table 2:** Wilcoxon signed rank test between intake and each of the follow-ups

Outcomes	p-value
**BDI**	

Difference between baseline and t2	0.0179

Difference between baseline and t3	0.0042

Difference between baseline and t4	<0.0001

**PCL**	

Difference between baseline and t2	0.0061

Difference between baseline and t3	0.0016

Difference between baseline and t4	<0.0001

**Reexperiencing subscale**	

Difference between baseline and t4	0.0020

**Avoidance subscale**	

Difference between baseline and t4	0.0123

**Hyperarousal subscale**	

Difference between baseline and t4	0.0043

BDI: Beck Depression Inventory

PCL: PTSD Checklist

**Table 3:** Effect sizes and changes in clinical outcome from baseline to final visit (N = 27)

	Intake (t1)		3-month follow-up (t4)		
	**Mean (*SD*)**	**Median (range)**	**Mean (*SD*)**	**Median (range)**	**Effect size**

BDI Total	30.44 (*7.85*)	29 (14 - 44)	20.67 (*10.05*)	19 (3-43)	1.09

PCL Total	56.11 (12.66)	55 (34-87)	46.85 (13.53)	46 (23-67)	0.90

PCL Reexperiencing	15.00 (*4.42*)	15 (7-25)	12.61 (*4.72*)	12 (5-22)	0.57

PCL Avoidance	23.40 (*5.22*)	23 (14-34)	21.05 (*6.02*)	21 (10-31)	0.38

PCL Hyperarousal	16.04 (*4.02*)	16 (10-22)	13.96 (*4.32*)	14 (5-21)	0.48

BDI: Beck Depression Inventory

PCL: PTSD Checklist

Cohen's d was calculated for effect size

The number and percentage of responders at each of the follow-ups are reported in Table 4. Thirty seven percent (10/27) were considered responders, as defined by a decrease in total PCL scores of at least 20 percent and for depression, 19% (5/27) were considered responders as defined by a decrease in total BDI score of at least 50%. Overall, a higher percentage of participants met criteria for being a responder on the PCL than the BDI.

**Table 4:** Frequency of responders at each of the follow-ups

Measure	Responders (%)		
	**1-month (t2) (n = 27)**	**2-months (t3) (n = 27)**	**3-month (t4) (n = 27)**

PCL	7(25.93%)	9(33.33%)	10(37.04%)

BDI	1(3.70%)	4(14.81%)	5(18.52%)

Responders on the PCL were those with 20% or greater improvement

Responders on the BDI were those with 50% or greater improvement

Additional analyses to examine the efficacy of aripiprazole among those with severe depression (BDI > 29) at intake (n = 14) found significant reductions in PCL symptom scores for this subsample as well, with the PCL scores averaging 61.79 (SD = 9.60), 51.86 (SD = 13.54), 52.14 (SD = 15.53), and 48.71 (SD = 13.02) at intake and each of the follow-ups, respectively. The Wilcoxon signed rank test results showed significant reductions from intake to each of the follow-ups (p = 0.0051, p = 0.0405, and 0.0010 for each of the follow-ups). The percentages of responders on PCL at each of the follow-ups are 35.71%, 42.86%, and 57.14%, respectively. It is worth noting that at t4, having severe depression was found to be associated with higher percentage of responders on PCL (P = 0.0461 from Fisher's exact test).

## Discussion

Consistent with previous studies in veterans, [32-34] the addition of aripiprazole contributed to a reduction in PTSD symptomatology in all symptom clusters (reexperiencing, avoidance/numbing, and hyperarousal) and among those with severe comorbid major depression disorder (baseline BDI ≥ 29). A significant number (37%) demonstrated a significant reduction in PTSD symptomatology (decrease in total PCL scores of at least 20%). Also consistent with studies on treatment resistant depression, [53,54] the addition of aripiprazole demonstrated an overall reduction in depression severity from a total BDI score of 30.44 at baseline to 20.67 at three months.

The low response rate of PTSD symptoms observed in this chart review, when compared to the augmentation trial by Roberts and colleagues [33], might be related to higher rates of comorbid depression (100%) in this patient group. Furthermore, unlike the study by Berman [55] where 42% met the criteria for significant decrease in depression severity, in our chart review only a minority (19%) showed decrease in total BDI scores of at least 50%. The low response rate observed in our chart review compared to Berman and colleagues' [55] study might be related to the fact that in Berman's study, patients with comorbid PTSD were excluded. Also in this chart review, most patients (52.9%) reported depression in the severe range and most patients (*n *= 18, 66.7%) in this sample continue to be symptomatic despite being prescribed combination antidepressant prior to initiating aripiprazole, suggesting significant treatment resistance. Although modest, the response rate is particularly encouraging in a population of chronic military-related PTSD with comorbid major depression in the severe range, which has traditionally demonstrated poor response to pharmacotherapy. Clinically, it stresses the importance of encouraging patients to persist with treatment and to consider augmentation strategies for patients who demonstrate a partial response (25-50% improvement) or no response with optimization of monotherapy.

Due to significant limitations of this chart review, including a small sample size, the retrospective nature of the design, and a lack of a control group, careful interpretation of the findings is warranted. This was a preliminary, open-label retrospective chart review, with a small sample size. However, since most patients in this chart review had PTSD for more than 10 years, with comorbid major depression in the severe range, the observation that the majority of patients had improved scores for both PTSD and depression severity is noteworthy.

## Conclusions

Military-related PTSD often presents with comorbid major depression requiring prompt and effective treatment. This chart review demonstrates that the addition of aripiprazole can assist in providing symptom relief in a population that has traditionally demonstrated poor pharmacological response. Further studies including double-blind, placebo-controlled studies are necessary to confirm our findings and further demonstrate the benefit of aripiprazole augmentation in the treatment of military- related PTSD.

## Competing interests

Drs. Richardson, Fikretoglu and Liu have no disclosures to announce in association with the contents of this issue. Dr. McIntosh has acted as a presenter for, participated on an Advisory Board for, and/or received research funding from Pfizer, AstraZeneca, Eli Lilly, Biovail, Bristol Myers-Squibb, Lundbeck, Forest, Servier, Sanofi-Aventis, Shire, and Janssen-Ortho.

## Authors' contributions

DR conceptualized and designed the chart review and drafted the manuscript. DF contributed to the statistical analysis and AL completed the statistical analyses. DR, DF, AL and DM contributed to the interpretation of the results. All the authors contributed to the preparation of the final manuscript.

## Pre-publication history

The pre-publication history for this paper can be accessed here:

http://www.biomedcentral.com/1471-244X/11/86/prepub
